# Perceiving the poetic world: A corpus-assisted transitivity analysis of poetry comics

**DOI:** 10.3389/fpsyg.2022.1061169

**Published:** 2022-12-28

**Authors:** Shukun Chen, Zenan Zhong

**Affiliations:** School of Foreign Languages and Cultures, Guangdong University of Finance, Guangzhou, China

**Keywords:** comic adaptation, poetry comics, intersemiotic translation, transitivity, process type

## Abstract

While modern adaptations of Chinese classics have drawn keen scholarly interests lately, the comic adaptation of Chinese traditional poetry remains under-investigated. Extending the previous research on intersemiotic translation and comics, this paper, drawing on the analytical framework of systemic functional semiotics, examines distribution of process types of language in poems in comparison with that in comic images in the exemplary case drawn by Cai Zhizhong, using UAM image as the annotation tool. The comic book formulates a multimodal corpus that consists of 1,097 clauses and 605 images. We have manually analyzed the process type of the poems and their corresponding comic panels. Our quantitative and qualitative results show that there are distinct patterns of process-type distributions between verbal poems and images. Poems have been turned into perceptions, actions, and verbal processes in comic strips, which serve various purposes such as construction of the poet’s gaze, relations building, storyline development, dramatization, metaphor visualization, etc. The paper is concluded with discussion on how the intersemiotic translation of poems might produce effects on readers.

## Introduction

Chinese poetic tradition has been one of the longest traditions in the world literature and it has continuously thrived not only in China but also in its neighboring countries and the western countries (Hinton, 2014, p. 1). To make the poetry more accessible to the general public, certain adaptation or translation are needed. One solution is to translate it into modern plain language, an act of intralingual translation. Another approach, as is increasingly well received, is to reconstruct it into a multimodal text, that is, poetry with pictures, the process of which is called intersemiotic translation in Jakobson’s (1959, p. 261) term. The appeal of adaptations, argues Hutcheon (2012), “lies in their mixture of repetition and difference, of familiarity and novelty (114).” Thus new forms of poem picture books have never ceased to emerge in recent years to meet the needs of readers of different ages and various kinds (c.f. Zhong et al., 2021a). Among these forms, comic adaptations have stood out as a peculiar type of innovation. The most successful case is Cai Zhizhong’s (or Tsai Chih Chung, hereafter Cai) comic adaptation of Chinese classics. Most remarkably, he managed to adapt Chinese classical poems into comics, which is rarely seen in poetry literature. However, his work of comic adaptation from poems has not been investigated, though there are a few scholars who have conducted scanty research on Cai’s comic adaptation of Chinese classics (e.g., Yu, 2019 on *Journey to the West*; Zhong et al., 2021b).

To better understand the phenomenon of intersemiotic translation from poems to comic images, we draw upon the analytical framework outlined in systemic functional semiotics with a specific focus on transitivity systems (Kress and van Leeuwen, 2006; Long and Peng, 2012; Painter et al., 2013; Halliday and Matthiessen, 2014) to examine the semiotic distributions in both verbal and visual resources in Cai’s case. The paper will first review relevant studies on intersemiotic translation and comic adaption. Then, the analytical framework of transitivity systems will be introduced before reports on how verbal poems were translated into images quantitatively and qualitatively. Then, we will consider how the intersemiotic translation strategy may produce discursive effects on readers.

## Intersemiotic translation and comic adaptation

The phenomena of various forms of adaptation such as film adaptation from literary works, comic adaptation from classics, etc., have been brought to the attention of translation scholarship under the concept of intersemiotic translation. The most cited source of the notion is Jakobson’s (1959, p. 261) tripartite definition of translation, namely, intralingual translation, interlingual translation, and intersemiotic translation. Intersemiotic translation, in Jakobson’s sense, refers to translation from language to other semiotic systems. Subsequent research attempted to broaden this concept to include translation from one semiotic system to another, not necessarily involving language (e.g., Eco, 1979, p. 71; Dusi, 2015; Mossop, 2019). Intersemiotic translation has been modeled theoretically in different terms such as transposition (Dusi, 2015), and resemiotization (Iedema, 2003; O’Halloran et al., 2016), giving rise to multimodal perspectives in translation studies, or more concisely termed, multimodal translation (c.f. O’Sullivan, 2013; Pérez González, 2014; Zhang and Feng, 2021) or plurisemiotic practices in translation (see Mus, 2021). Given the complexity of expression planes and content planes of two semiotic systems, intersemiotic translation inevitably introduces creation and discrepancies, and may differ from traditional notion of translation by nature. Nevertheless, the notion of faithfulness to the source text remains the central issue in studies on adaptation strategies (e.g., Hutcheon, 2012; Roche et al., 2018). Therefore, comic adaption can also be investigated in terms of faithfulness and transformation to the original verbal text, shedding some light on functional and cultural motivations.

Early research on translation of comics mostly revolved on how multimodal features of comic discourse motivated translation choices (e.g., Kaindl, 1999; Zanettin, 2008). As Borodo (2015) suggests, the interaction between verbal and visual sign systems on comic pages may be pertinent to translation for reasons such as spatial constraints, verbal-visual incongruence, liberal interpretation of a panel, etc. Recently, there has been a proliferation of studies that investigated how classic texts were translated inter-semiotically into comics and picture books. Some studies are interested in the translation shifts and the motivations underlying these choices. For instance, Chen (2018) found that the visual translation of Mulan has been “transformed from a legendary Chinese heroine into a hybrid Americanized tomboy.” Other research focuses on equivalence such as Luo (2021), who investigated how verbal rhetorical figures correspond to visual ones in a picture book version of *Art of War*. However, how poems could be visualized into comics has not received much scholarly attention. And the previous studies showed no attempt in drawing on linguistically-minded theories for systemic and quantitative analysis for the semiotic shifts in translation. Therefore, the current study attempts to contribute to this area by analyzing how the experiential meanings of verbal poems were transformed into comic images under the framework of systemic functional semiotics.

In recent years, systemic functional linguistics has already been employed to investigate how classic literary texts are transformed lexicogrammatically and semantically in adapted versions. For example, Huang and Chen (2015) conduct functional linguistic analyzes of the beginning paragraphs of Alice’s Adventures in Wonderland and the corresponding simplified versions. Chang (2018) treats different versions of *Pride and Prejudice* as “re-instantiation” and analyzes how the targeted texts are interpersonally and ideationally committed. While these functional studies are concerned with English literary texts, the Chinese classics and their adapted texts remain largely under-investigated. Chen (2018) pioneers this field by comparing the transitivity, projection, and appraisal of the classic Chinese fables and their modern adaptations to reveal how social and semiotic developments might influence the latter. But the study is confined to linguistic analysis. A few scholars have begun to use systemic functional semiotics to investigate comics (e.g., Bateman and Veloso, 2003; Veloso and Bateman, 2013; Wang 2014; Zeng and Yang, 2016). Veloso and Bateman (2013), for example, conduct a multimodal examination on how legitimization has been constructed in Marvel’s Civil War comic books. Zeng and Yang (2016) attempt to establish a framework describing how projection (language of speech) could be realized in comics. Despite the keen interests of functional linguists in both adaptations and comics, few of them are concerned with intersemiotic translation in comic adaptations. And it remains a question how poems could be translated into other semiotic systems such as images in picture books and comics. Therefore, this study is an attempt to address the following questions:

1. How are poems translated/resemioticized into comic images?
2. How are the process types distributed in poems in comparison with those in comic images?
3. What are the functions of the processes in images with reference of those in poems?

## Theoretical framework and methodology

**Figure 1:**
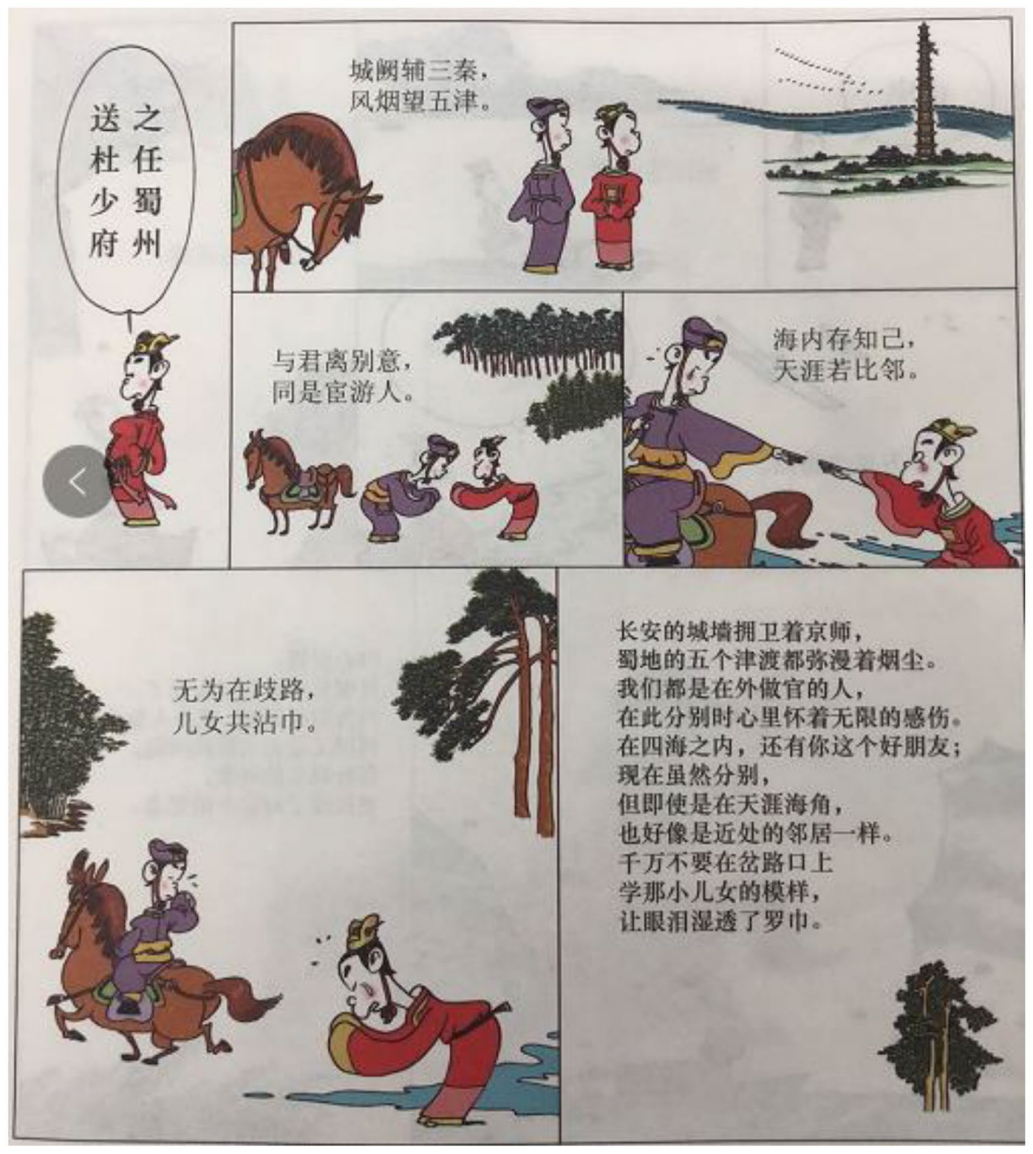
A complete poetry comic strip in Cai (2014, p. 3). Images reproduced with permission from Shang Dong People Press.

In this paper, we focus on a particular case of Cai’s adaptations, namely, *Tang Shi San Bai Shou: Qian Gu De Jue Chang* (300 Tang Poems: Songs of millenniums). There are a few features in this book that we should mention here. First, there are 140 poems selected into this book although the title claims that there are three hundred. Thus, the number “three hundred” should not be interpreted literally as is often the case in other anthologies of Tang poems. Why these 140 poems have been chosen is not mentioned in the book. But the poems, as indicated in the introduction of the book, are among the most popular ones. Second, each poem is transformed into a comic strip of, in most cases, six panels. Usually, a panel displays one or two lines of the corresponding poem. Third, the general structure of the comic strip is rather fixed. The first panel presents the title of a poem through a speech bubble. Then, the following four panels demonstrate the body of the poem. The last panel provides the explanation of the poem in plain modern Chinese (see Figure 1).

To get quantitative results, we worked under the general framework of systemic functional grammar and the visual grammar that was developed based on systemic functional linguistics, both of which could also be referred to as systemic functional semiotics (or SFS, see, e.g., Bateman, 2015) or social semiotics (see, e.g., Jewitt et al., 2016, p. 58–85). Since Halliday (1978) outlined his position to study language from a social semiotic perspective, the paradigm of the linguistic theory has been effectively applied to the analysis of other semiotic modes such as images, sound, actions, etc. (Martinec, 1998; Van Leeuwen 1999; Kress and van Leeuwen, 2006; Painter et al., 2013; respectively). There are a few basic assumptions made in systemic functional linguistics which seem to work for other semiotic resources as well. One of the most significant hypotheses is that language has become the way it is because it serves some basic functions that help human beings relate themselves to the ecological and social environment. All these functions are general enough to cover certain clusters of lexicogrammatical systems. The functions are boiled down into three metafunctions, namely, ideational function, interpersonal function, and textual function.

This paper focuses on ideational function, and more specifically, the transitivity system that serves that function. Ideational function means that language helps human beings to communicate and reflect on the physical and abstract world. It provides a theory of human experience (Halliday and Matthiessen, 1999). This function is mainly grammaticalized into transitivity system in which clause components are labeled as participant, process, and circumstance. Process is the core element that determines the configuration of participants and circumstances. Thus, in each clause, we can identify six types of process including material process, mental process, relational process, existential process, verbal process, and behavioral process. The type of process determines how other participants are configured in a clause. For example, a material process may go with a goal (as in *He drove a car*) and a verbal process may go with a metaphenomenon (as in *He says a car is coming*). But it is not possible for a material process to go with a metaphenomenon (*He drove a car is coming). Therefore, a transitivity analysis reveals how a world is depicted experientially in a particular text. Since the source text is Chinese, we mainly drew upon Long and Peng’s (2012) transitivity system in Chinese to annotate the process types in verbal poems. The criteria for coding the process types in language are listed in Table 1. In cases where double coding may apply, the authors will reconcile with each other to determine the particular process in a given clause.

**Table 1:** Coding criteria for process types in language.

Process types in language	Coding criteria
Material process	Processes of doing and happening, e.g., creating, cutting, walking, etc.
Mental process	Processes of our consciousness, e.g., thinking, seeing, feeling, etc.
Relational process	Processes of characterizing or identifying, e.g., being, having, etc.
Verbal process	Processes of saying
Existential process	Processes that represent that something exists or happens, e.g., there be
Behavioral process	Processes of (typically human) physiological and psychological behavior, like breathing, coughing, smiling, dreaming and staring.

**Figure 2:**
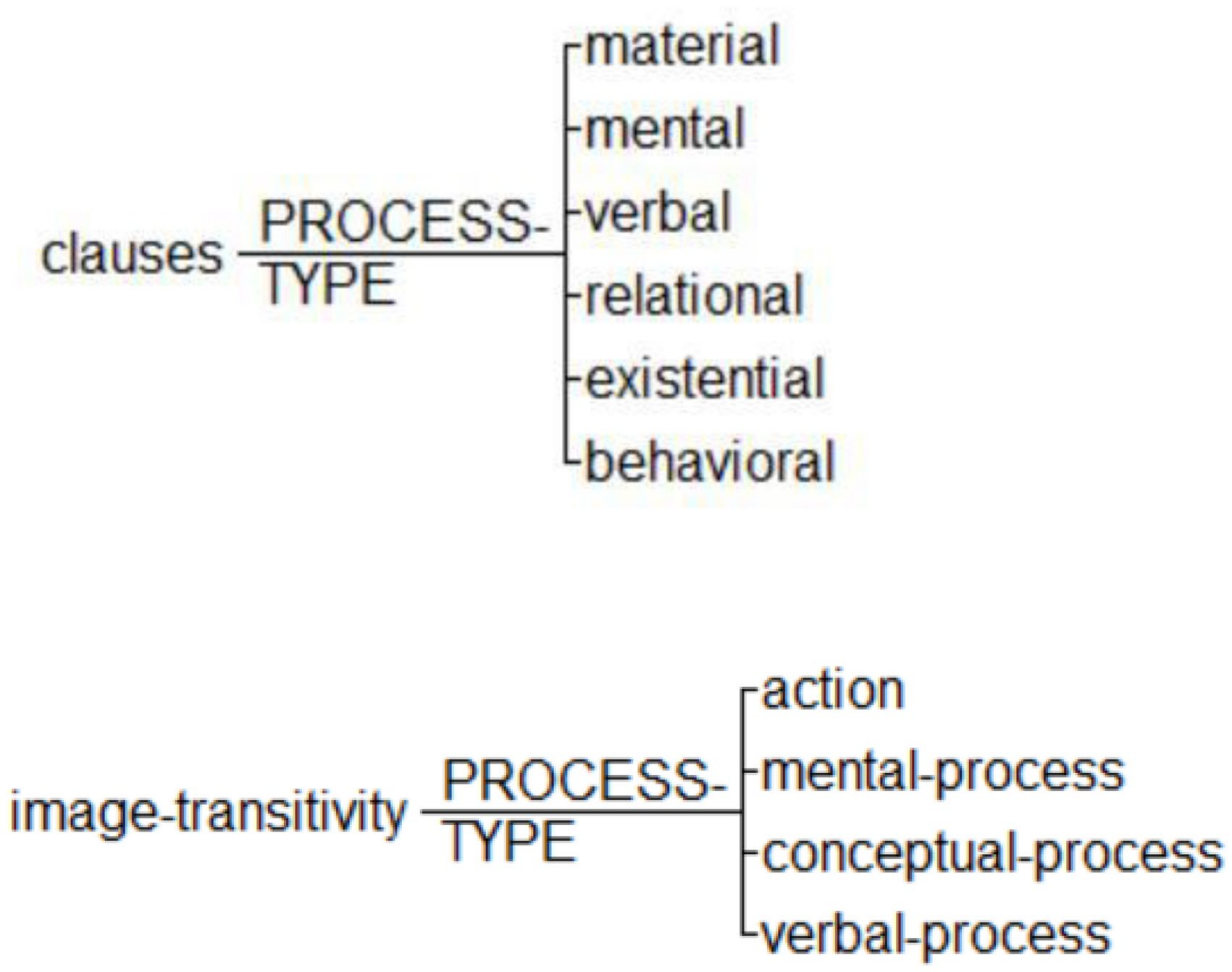
Annotation schemes for process types in poems in parallel with process types in images.

**Figure 3:**
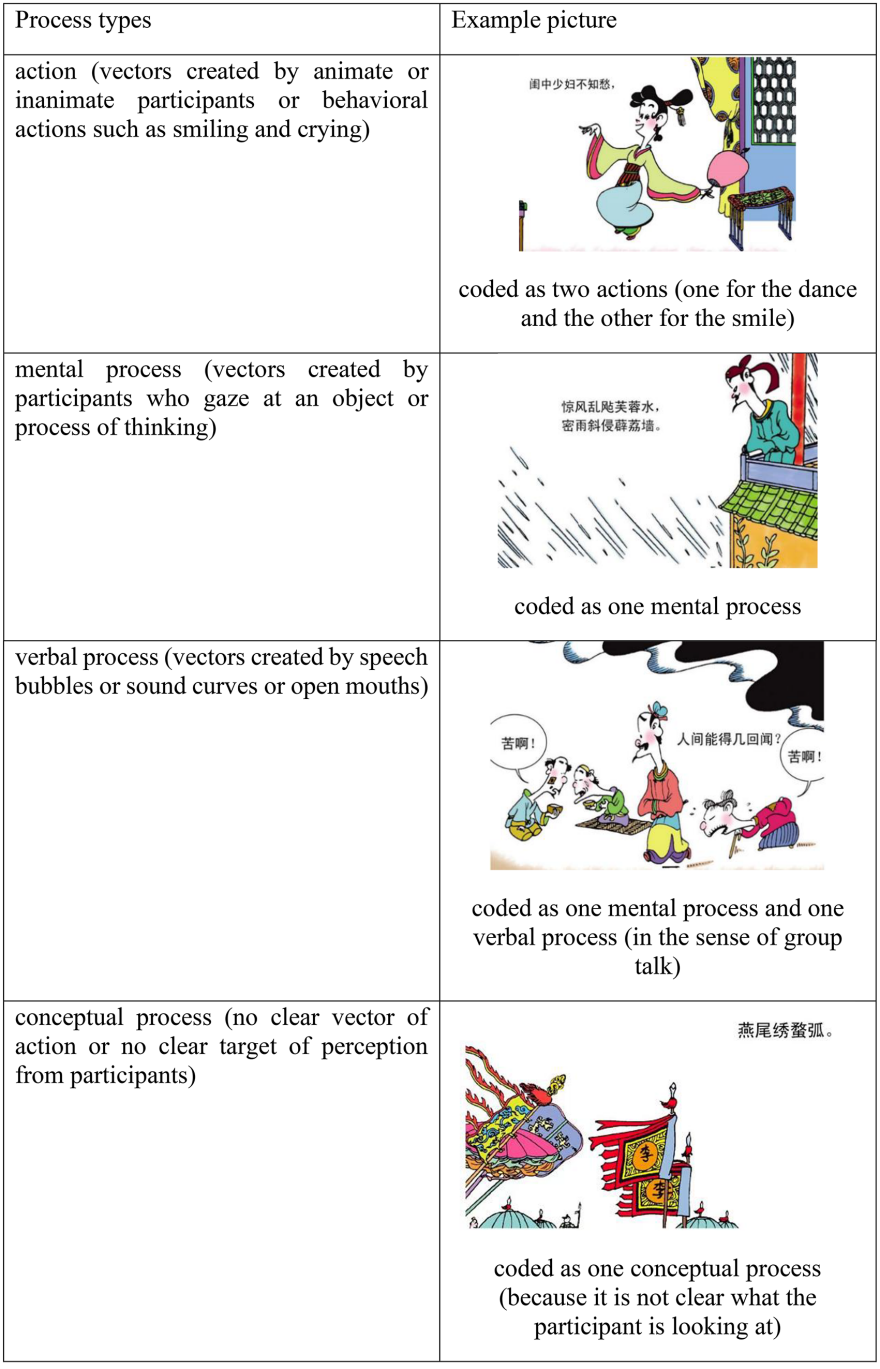
Process types in images following Painter et al. (2013, p. 69). Images reproduced with permission from Shang Dong People Press.

The transitivity system in language has been applied to visual analysis by Kress and van Leeuwen (2006) and Painter et al. (2013). In this study, we mainly adopt the model of Painter et al. (2013). In their framework, the transitivity system of images is described in terms of participants, processes, circumstances, and their inter-relations. We follow specifically how they model different processes, or in Kress and van Leeuwen’s (2006) term, narrative processes (see Figure 2). In this framework, there are three major types of processes, namely, Action, Verbal Process, and Mental Process. Notice that these processes are all featured with clear vectors (for example, directions of actions or gazes). When there is no vector, the process will be coded as Conceptual Process, following Kress and van Leeuwen (2006). Hence, we have a list of process types which are comparable between languages (the poem) and images (the comics), as shown in Figure 3.

**Figure 4:**
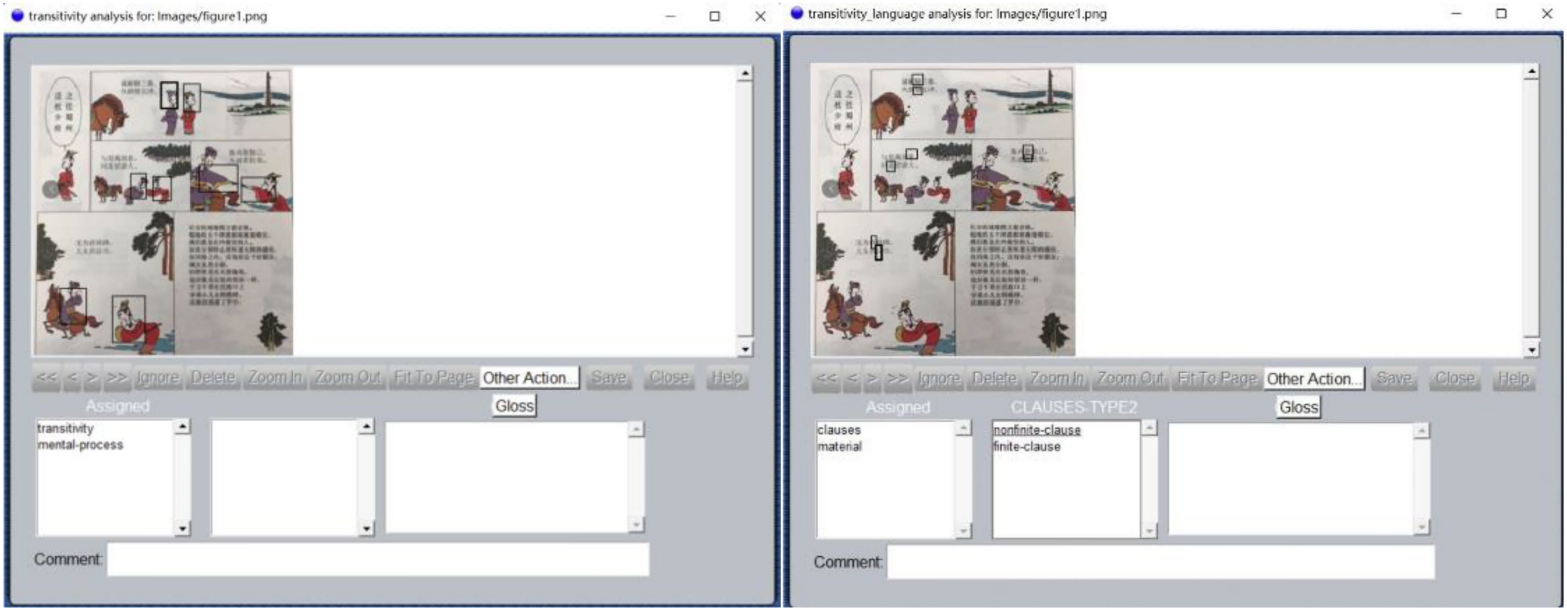
Annotating the images (left) and language (right) of the comic strips (Cai, 2014) with UAM Image Tool 2.1. Images reproduced with permission from Shang Dong People Press.

With the frameworks mentioned above, we scanned the pages and imported them in UAM Image Tool for annotation (see Figure 4). We manually annotated the process type of each clause in poems and each panel of the comic strips in our case. The comic book generated a small multimodal corpus of 1,097 clauses and 604 images (excluding the first and last panels of each comic strip because they manifest the same content repeatedly and thus would affect our analysis on patterns of transformation). The two authors of this paper conducted a pilot coding of the first 20 clauses and the corresponding images and discussed the discrepancies. Then, the two authors agreed that a few adjustments were needed to tackle the peculiar features of our data. The following criteria are relatively more operable.

First, whether there is a clear vector formulated in the image is the defining feature to separate narrative image from conceptual image (following Kress and van Leeuwen, 2006). When it is judged as a narrative image, we then decide whether the vector is created by actions, mental processes, or verbal processes. Next, the number of these processes will be counted according to the number of entities that produce the vector. Take Figure 4 for example. In the second panel, two men are portrayed as looking at a pagoda on the right side. Based on the vector of gazing and the number of men, we coded this image as two mental processes. However, when a certain object serves as the tool with which the action is carried out, we would not count it in. The reason is that actions of tools should be considered as minor processes rather than major processes (like circumstances in a clause in language, c.f. Halliday and Matthiessen, 1999, p. 217–222). For instance, in Figure 4, the movement of the horse would not be counted as action, but the man riding it would be counted. Moreover, collective actions (such as a group of soldiers fighting in a war) are counted as one action unless a few individuals are foregrounded in the image.

Secondly, conceptual process will be coded only when there is no vector involved in a comic panel. And the image will be coded as one conceptual process no matter how many elements or items are depicted in it. We took this approach because it was difficult to separate conceptual processes from various types of actions in one image, and it is both hard and unnecessary to count the number of conceptual processes. Consider Figure 4 again. If we coded the circumstantial elements such as the pagoda, horses, and trees as conceptual processes, that would have produced too many conceptual processes that are not significant to our analysis. And there is no point in counting how many trees and horses. Therefore, an image without clear vectors will be coded as one conceptual process.

Third, the acts of crying, smiling are coded as action as well, because they are coded as behavioral action in Painter’s et al. (2013) model. Furthermore, to address the particular nature of the verbal process in comics, the person who is depicted as saying something with the mouth open with or without the speech bubble would be coded as one verbal process. And the signal of any sound from animate objects (such as birds) or inanimate objects (such as trees or houses) is coded as verbal process as well.

Despite the adjustment of the coding criteria given above, the analysis of the language in Chinese and the images was inevitably subject to subjective interpretations due to the ambiguity in poetic language and visual meanings. Therefore, the two authors coded the data separately to reach inter-rater reliability. It should also be noted that the analytical framework offered above merely focused on the processes in language and images, which represent only one of the many dimensions of meanings, neglecting, for instance, interpersonal meaning, textual meaning, logical meaning, etc. The subsequent analysis thus only shines a light on how poems could be resemioticized into images from a limited perspective.

## Quantitative results

This section will show the statistical result of our quantitative analysis. Table 2 displays the percentage of various processes in poems. It is shown that material process accounts for nearly half of the total (48.4%), followed by relational process (22.33%) and mental process (18.23%). Existential process and verbal process both take up only around 5%. When it comes to processes in images, a clearly similar pattern can be perceived, as shown in Table 3. Mental processes occupy 46.85% of the total, followed by action (43.11%). Most notably, there is only one conceptual process recognized in our data. Verbal process takes up 9.91%. Hence, this preliminary comparison reveals that the relational and existential processes in poems have been shifted to action, mental processes, and verbal processes. The result is plausible because actions and verbal processes are the predominant semiotic resources used to construct events and narratives in comic strips. However, why mental processes were adopted to represent various processes in poems needs close examination. The next section will provide qualitative analysis on the functions of action, mental processes, and verbal processes in images with reference to the processes in poems.

**Table 2:** Distribution of different processes in poems.

Process types in poems	Count	Percentage
Material process	531	48.40%
Mental process	200	18.23%
Relational process	245	22.33%
Existential process	55	5.01%
Verbal process	54	4.92%
Behavioral process	12	1.09%
Total	1,097	100.00%

**Table 3:** Distribution of different processes in images.

Process types in images	Count	Percentage
Action	335	43.11%
Mental process	364	46.85%
Verbal process	77	9.91%
Conceptual process	1	0.13%
Total	777	100.00%

## Qualitative analysis

Based on the statistical analysis of our data, we examine, panel by panel, why certain processes in poems are resemioticized into other processes in images. As mentioned above, the general trend is that various processes in poems have been mostly reconstructed as mental processes, followed by actions and verbal processes. Hence, in this section, we will report on discursive features of mental processes, actions, and verbal processes in comic images and their functions, respectively.

### Functions of mental processes

**Figure 5:**
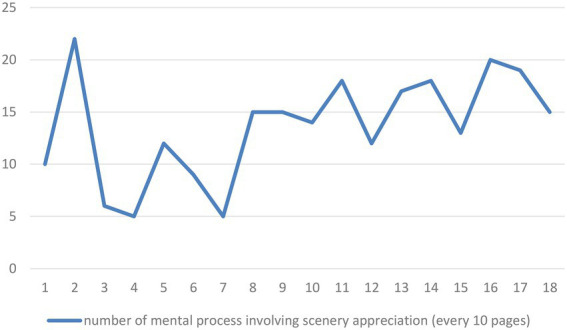
Number of visual mental processes involving perception by every 10 pages across the comic book.

A close examination of mental processes in images reveals that the most prominent motivation is that when the poem depicts objects or circumstances that are perceived, the perceiver tends to be foregrounded. In other words, the poet’s gaze has been constructed through mental processes of perception. Figure 5 displays the number of visual mental processes involving perception across the comic book.

Hence, it is conceivable that mental processes are often added in images, showing the poet or the protagonist observing the scenery in the poem. For example:

1.密雨 斜侵 薜荔 墙 heavy rain attacks creeping-fig wall.

“The heavy rain attacks the wall full of creeping figs.”

**Figure 6:**
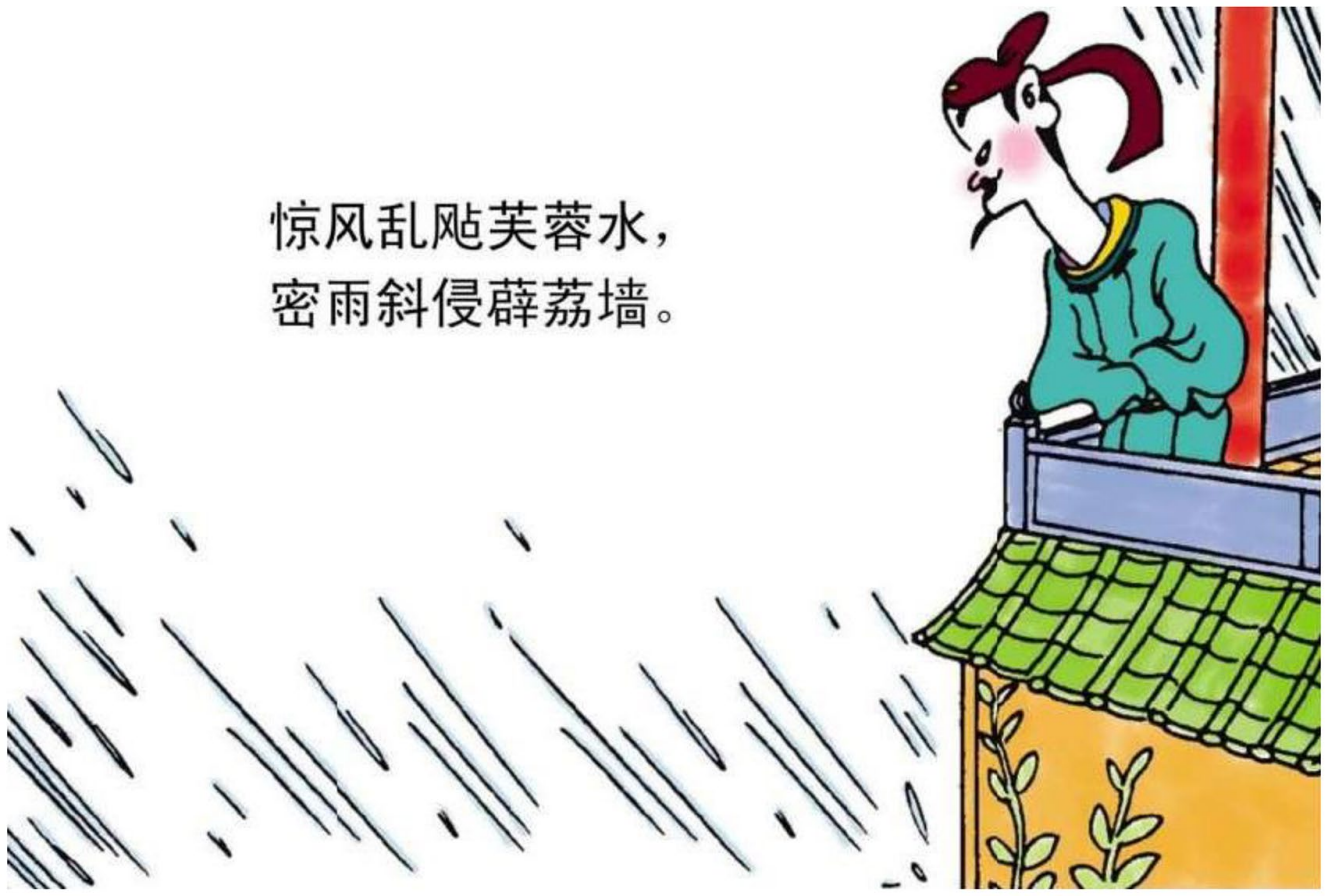
The third panel on Cai (2014, p. 116). Images reproduced with permission from Shang Dong People Press.

The image that depicts the line in example 1 not only displays elements of rain, creeping figs, and the wall, but also shows a man looking down at the rain on the wall (see Figure 6). This is the major reason why the mental process is the most frequent type in images. The poet or the protagonist of the poem is often foregrounded in the image to observe (or, in a few cases, think about) the elements or events portrayed in the poem.

The second motivation, which is less obvious, is to use mental processes of perception to reconstruct the relations between different elements in poems. More specifically, the vector of perception is utilized as a pointer to imply the relations. The relations might refer to more concrete sense of directions or more abstract meaning of interactive status. See the examples below.

2.皆 向 沙场 老 all toward battlefield old.

“(They) all get old along with the battlefield.”

3.联 步 趋 丹陛， together walk toward emperor.

分 曹 限 紫微 divide position only fate.

“(We) walk together towards the emperor.

Mere fate divides us into different positions.”

**Figure 7:**
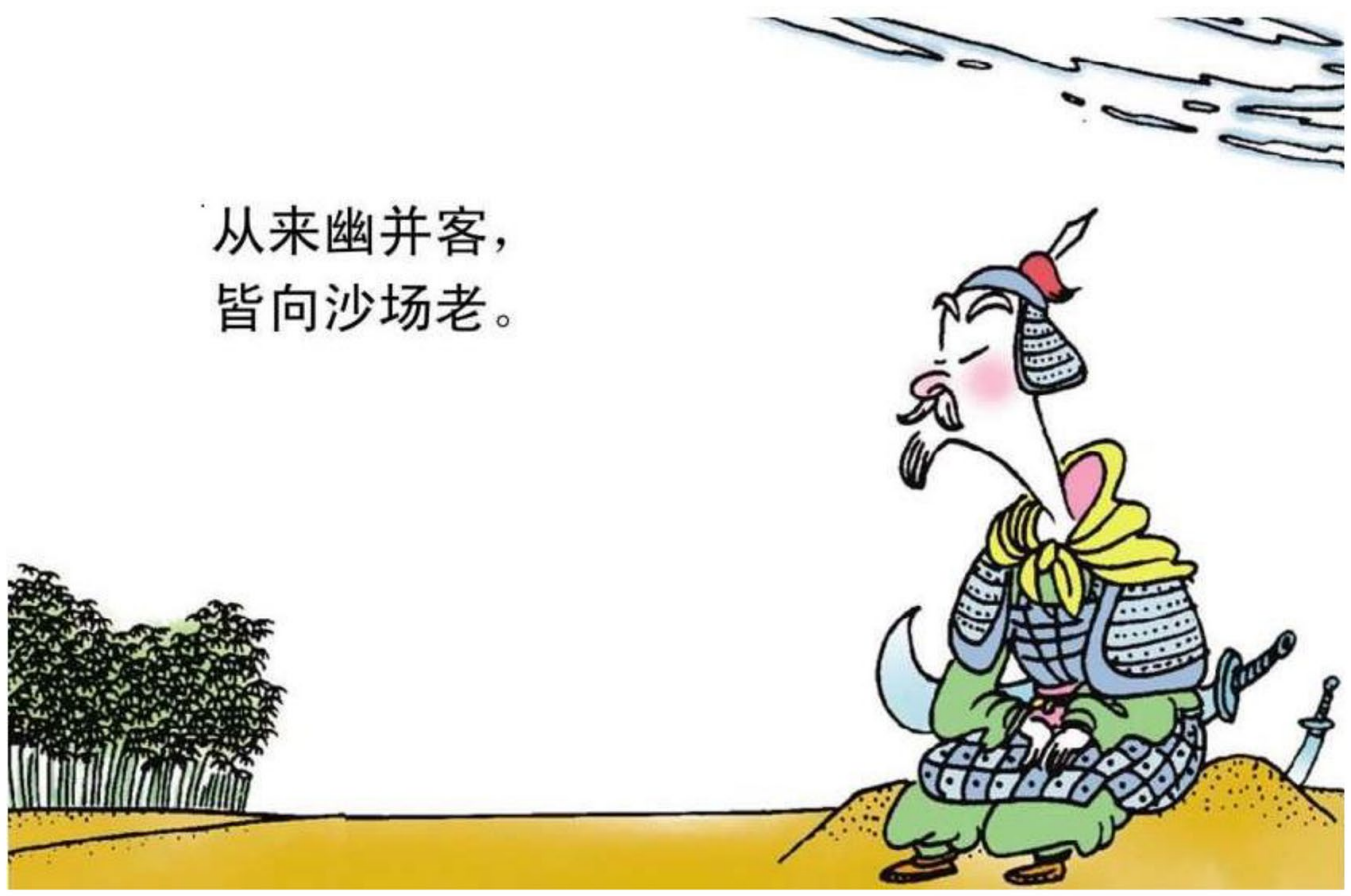
The forth panel on Cai (2014, p. 30). Images reproduced with permission from Shang Dong People Press.

**Figure 8:**
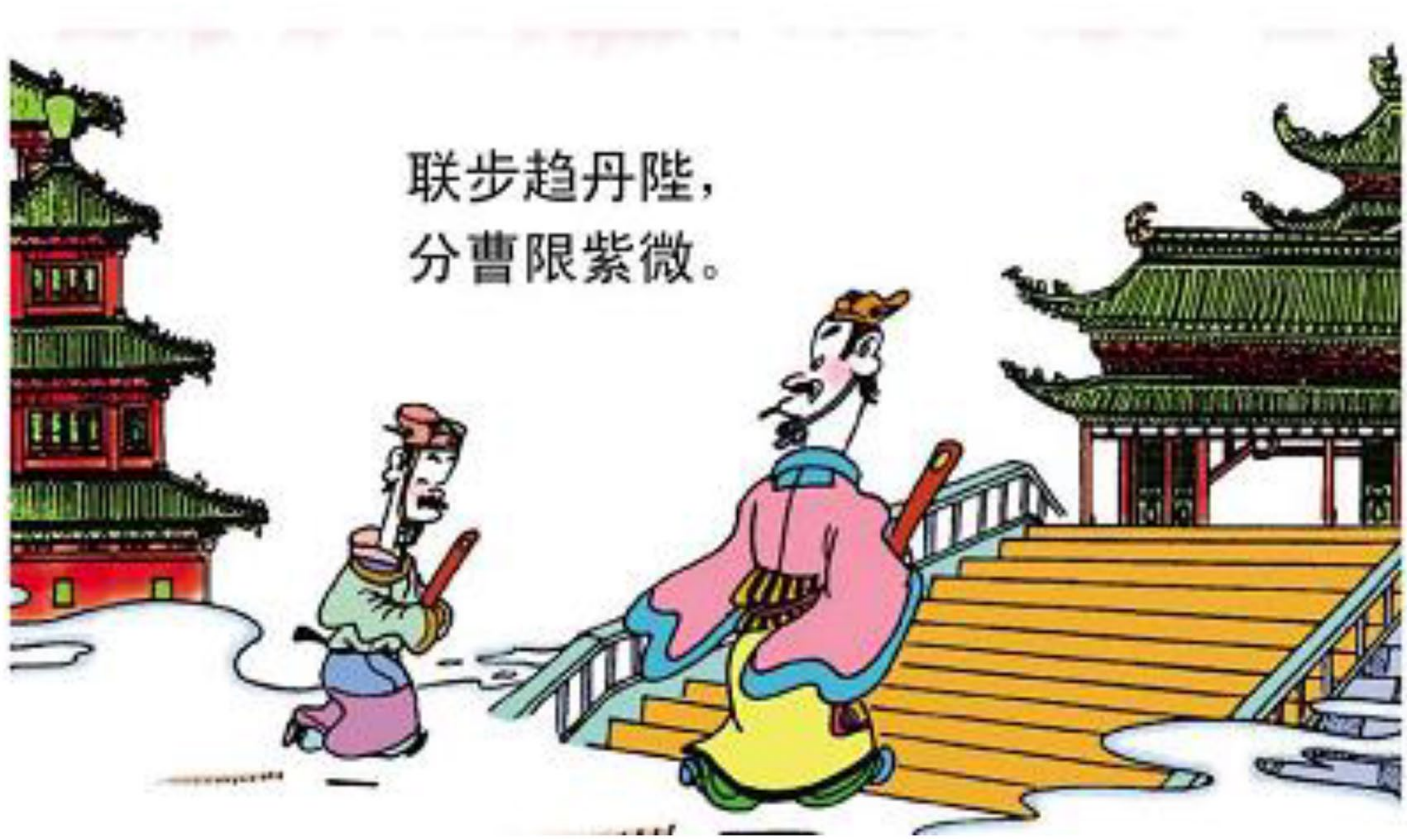
The second panel on Cai (2014, p. 68). Images reproduced with permission from Shang Dong People Press.

In example 2, the relational process 老 “old” is reconstructed as a soldier who is looking far into the battlefield. The vector of the gaze, thus refers to the relation between the man and the field (see Figure 7). In example 3, the material process 联步 “together walk” is turned into two types of processes in image, *viz.* action of walking and mental process of perception (see Figure 8). The perception suggests that the two men are connected in terms of their functions to serve the king. Therefore, the invisible line of vector serves to build up the abstract relations between the two protagonists in the poem.

### Functions of actions

**Figure 9:**
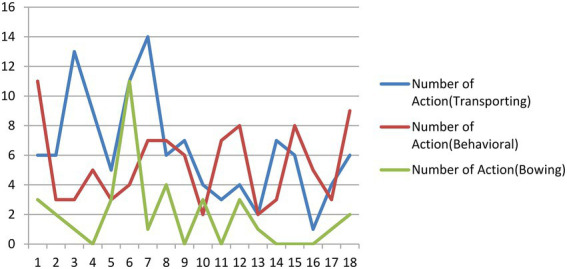
Number of actions involving transporting, behavioral and bowing by every 10 pages across the comic book. Images reproduced with permission from Shang Dong People Press.

As shown in the previous section, actions represent the second largest proportion in images. The most important function of actions in the poetry comic book is to construe the “come and go” of characters or poets. They might be portrayed as walking, riding a horse, or sailing on a boat. Figure 9 shows how these actions spread across the comic book. They are the most typical semiotic resources for the comic artist to (i) introduce the characters into the comic strip, (ii) reconstruct how people interact in poems, and (iii) develop the storyline in a comic strip (as exemplified in Figure 1). Figure 9 also shows the distribution of behavioral actions and actions of bowing (typically, two individuals bowing to each other).

**Figure 10:**
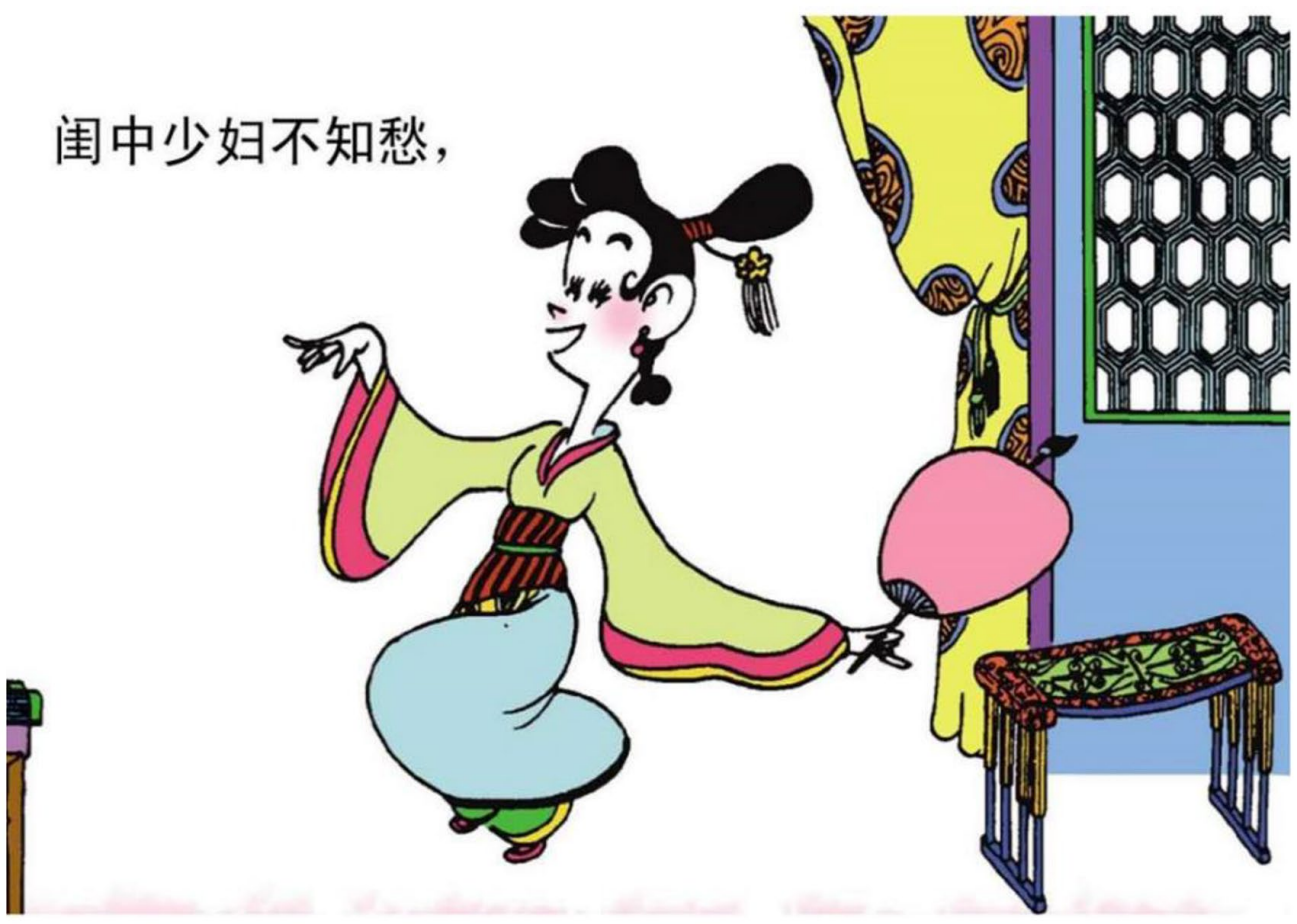
The second panel on Cai (2014, p. 33). Images reproduced with permission from Shang Dong People Press.

Apart from the actions of moving, we have also identified several important motivations for actions. The most obvious motivation is to construct affect and attitude in poems through actions, and more specifically, behavioral actions. This motivation could be best illustrated in examples 4 to 6 below. In example 4, the relational process 为乐 “be happy” is transformed into a smiling face in comics. In example 5, the process 若 “is like” is turned into the action of two men, with tears in their eyes, trying to reach for each other. In example 6, the mental process 不知愁 “does not know worries” is reconstructed as a jubilant dance as shown in Figure 10. Hence, action is an important resource for the comic artist to construct emotions realized directly or indirectly in poems. It is a truism that one of the most important functions of poems is to vent emotions. And it is difficult to visually express the delicate feelings construed in poetic language through comic images. The comic artist in our case is particularly adept at bringing the inner feelings out through facial expressions and actions.

4.烹 羊 宰 牛 且为乐， cook sheep cut ox to be happy.

“We are happy to cook sheep and ox”.

5.天涯 若 比邻 distance is like neighbors.

“We are like neighbors despite the distance between us.”

6.闺中 少妇 不知 愁.

in (her) bower a young wife not know worries.

“A young wife knows no worries in her bower”.

**Figure 11:**
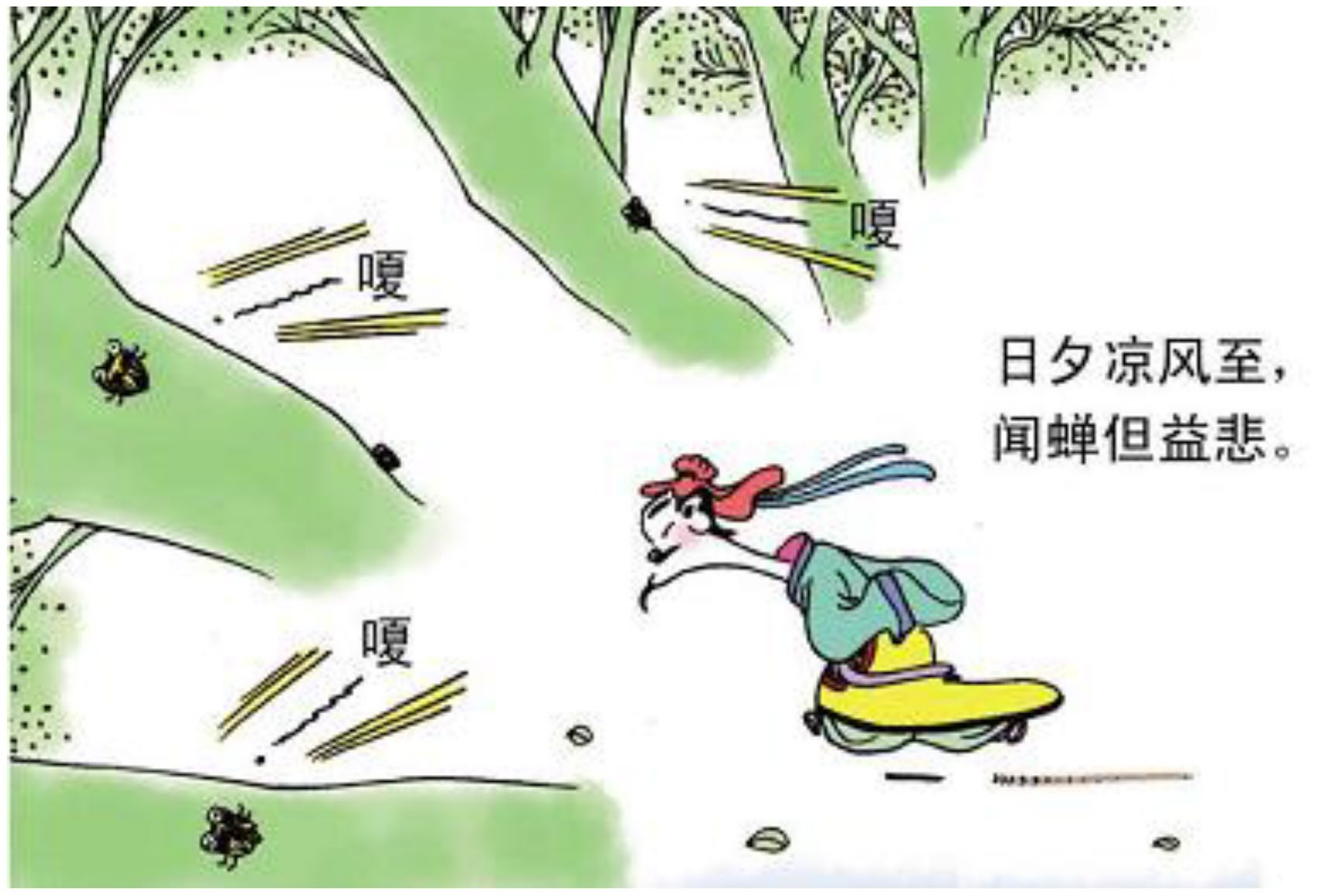
The fifth panel on Cai (2014, p. 21). Images reproduced with permission from Shang Dong People Press.

**Figure 12:**
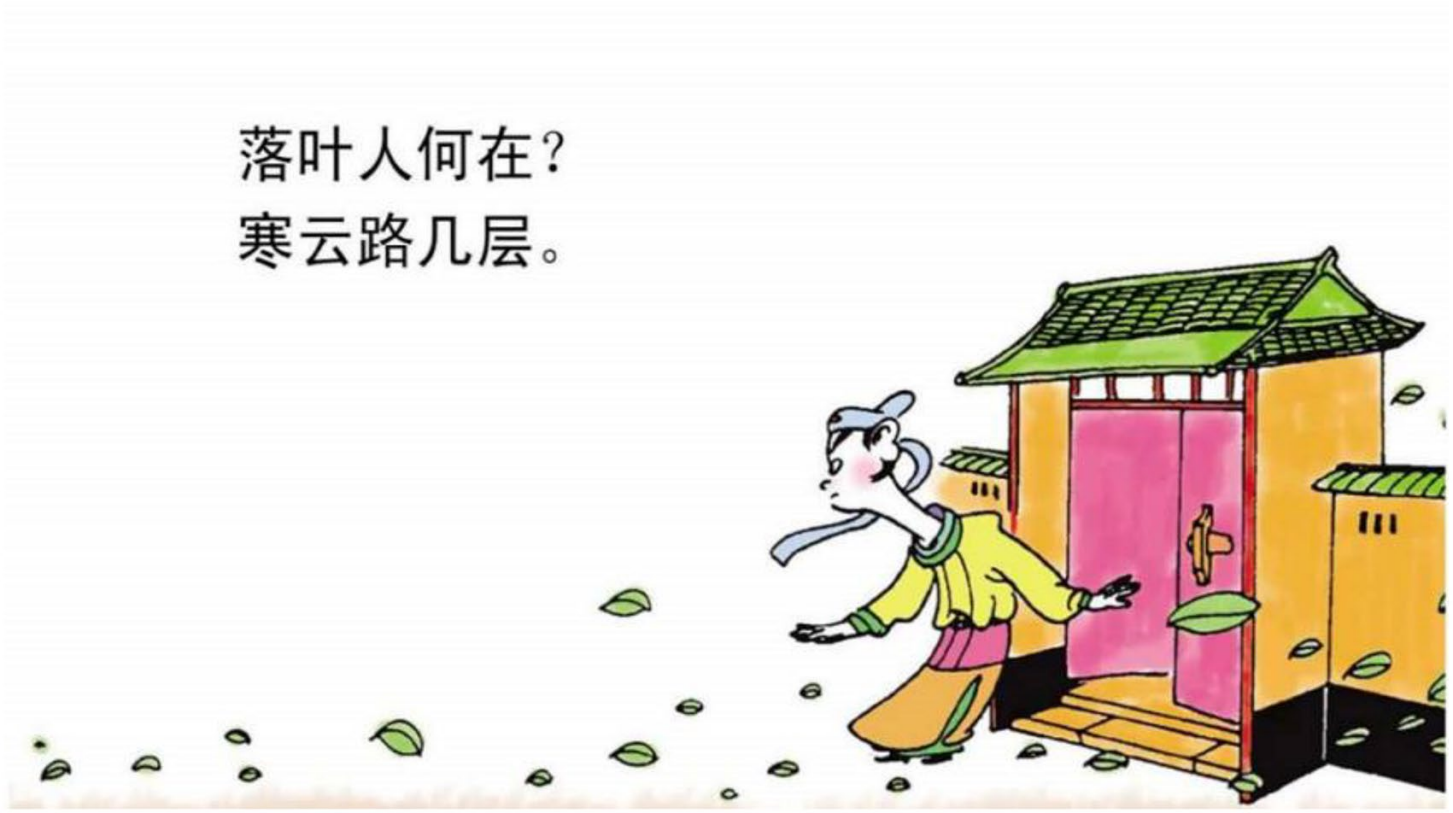
The third panel on Cai (2014, p. 143). Images reproduced with permission from Shang Dong People Press.

The second motivation that we find is to use actions to signal the change of cognitive status in poems. In this case, the actions of walking in or out of the panel are drawn as a way to represent the sudden change of knowledge or perception of the poet or the protagonist described in the poem. Consider example 7. The mental process 知 “know” in the poem refers to the mental status of the poet. In comics, the cognitive process is transformed into a man rushing out of a house, looking at the flowers. This action of moving into the scene indicates that the man does not know what has happened to the flowers. Similarly, in example 8, the mental process 闻 “hear” is turned into the action of a man walking swiftly toward the woods of multiple cicadas with parallel curvy lines coupled with Chinese characters indicating the sounds (see Figure 11). The action shows that the poet is rushing out in response to a sound, thus signaling the emotional change. In example 9, the action of stretching arms indicate that the poet suddenly realized there was nobody there to help (see Figure 12).

7.花 落 知 多少.

Flower fall know how many.

“(I) do not know how many flowers have fallen.”

8.闻 蝉 但 益 悲 hear cicada but more sad.

“(I) feel sad when hearing cicada.”

9.落 叶 人 何 在? Fallen leave person where.

“Where are the people when the leaves fall?”

The third motivation is to use actions to visualize metaphors in poems. It is not surprising that metaphors are frequently used in poems to describe things and circumstances that the poet comes to perceive. The comic artist may choose to transform the metaphor into actions. Consider the line in example 10.

10.落 花 犹似 坠楼人 falling flower be like falling people.

“The falling flowers are like people who fall from the mansion.”

11.汉文 有道 恩 犹 薄.

The Emperor Han Wen has a way grace still thin.

“The emperor Han Wen, though a good king, has no mercy.”

12.道 是 无晴 却 有晴 say be not sunny but sunny.

“It is said that it is not sunny but it is.”

**Figure 13:**
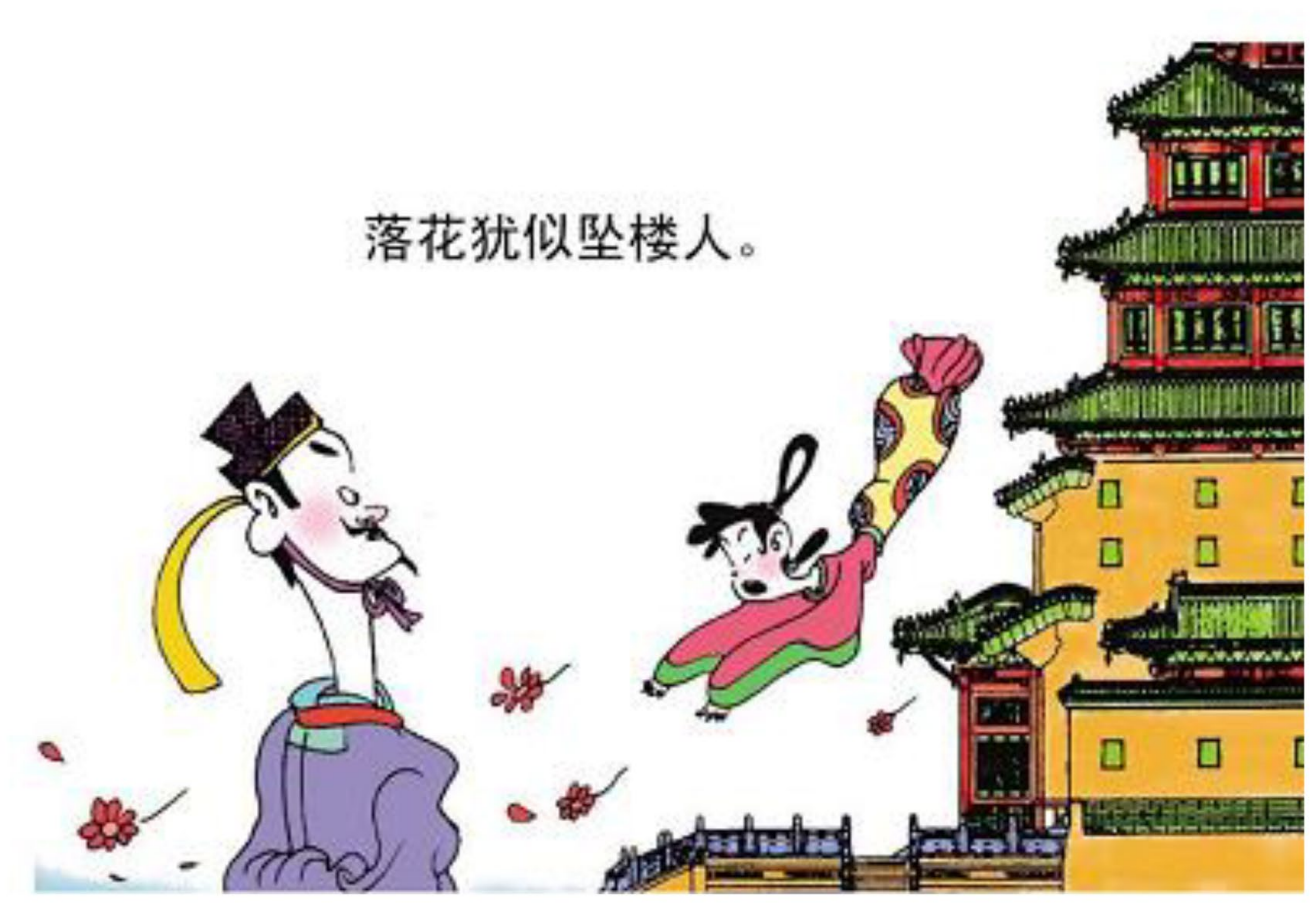
The fifth panel on Cai (2014, p. 136). Images reproduced with permission from Shang Dong People Press.

**Figure 14:**
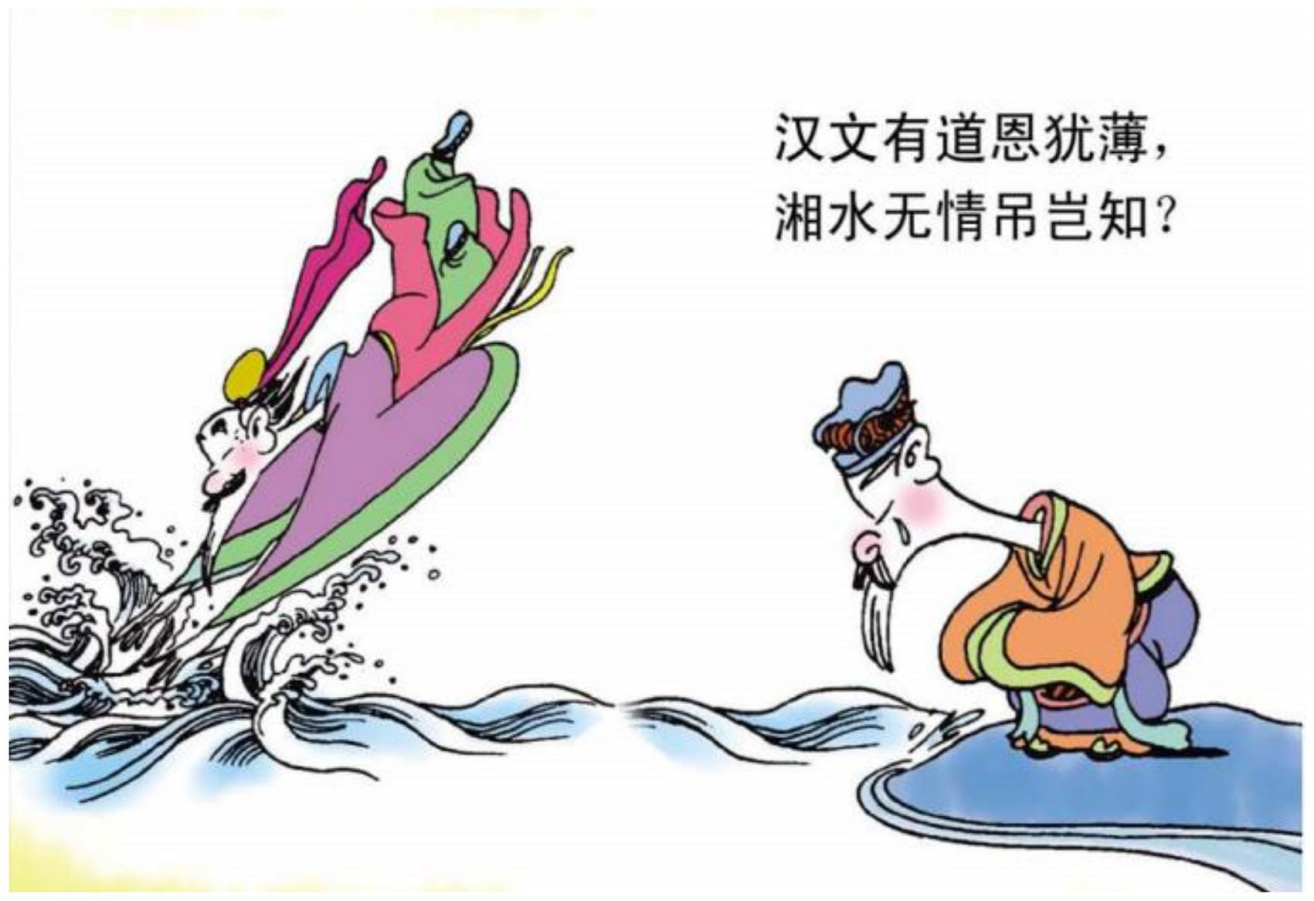
The forth panel on Cai (2014, p. 52). Images reproduced with permission from Shang Dong People Press.

**Figure 15:**
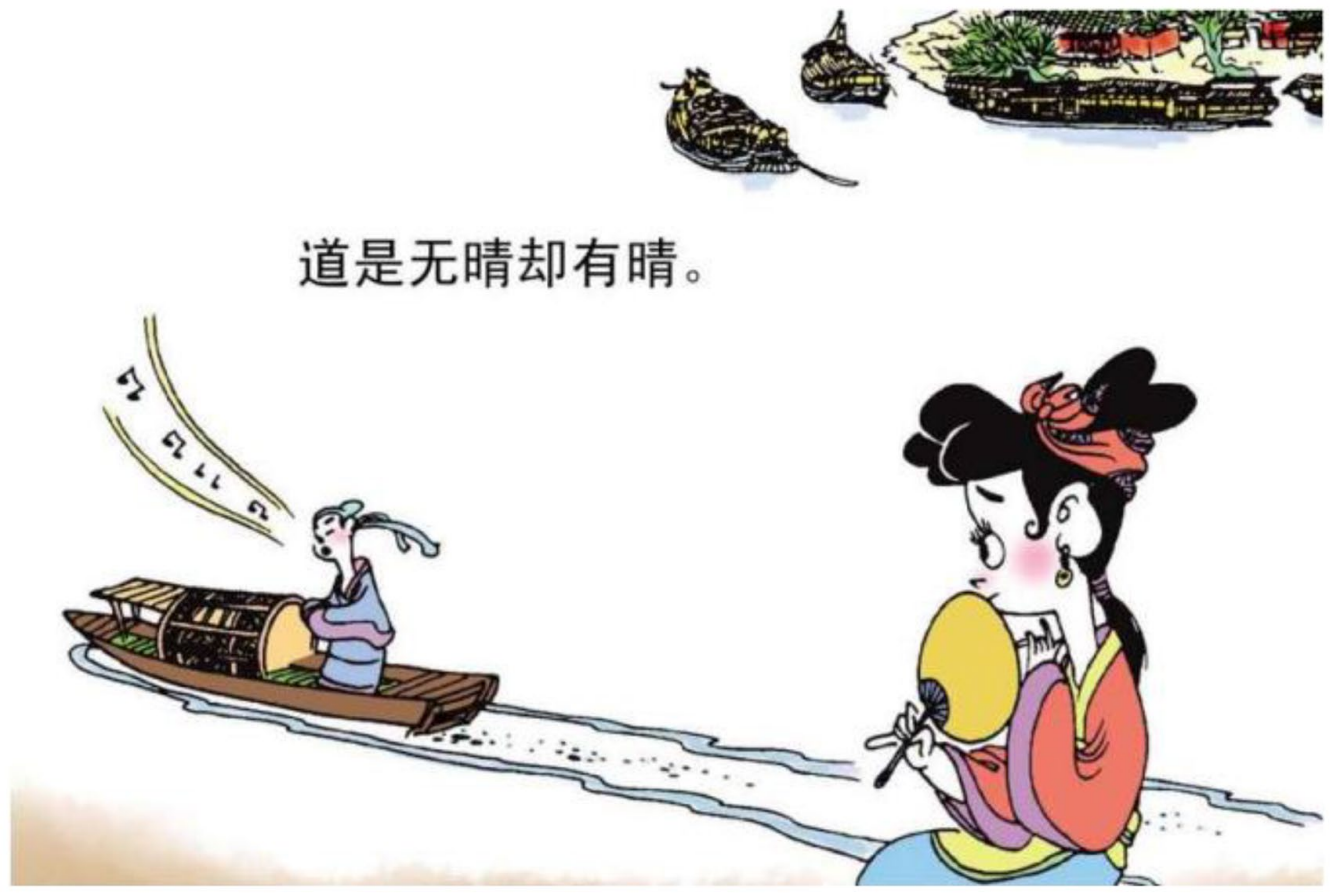
The fifth panel on Cai (2014, p. 99). Images reproduced with permission from Shang Dong People Press.

The relational process 犹似 ‘be like’ is reconstructed as a lady falling from a mansion (see Figure 13). Hence what should be considered metaphorical has become something perceivable. In example 11, the action of jumping into a river is used to symbolize the ruthlessness of the king (see Figure 14). In example 12, the relational processes 无晴 “not sunny” and 有晴 “sunny” are used to metaphorically express whether a lover is ruthless or sentimental. The author reconstructs that layer of meaning into a man singing on the boat (see Figure 15). The reader can therefore relate the action of singing and boating to the emotional feature of the man depicted in the poem.

13.忽 见 陌头 杨柳 色 suddenly see wild willow color.

“(She) suddenly sees the vigor of willows in the wild.”

**Figure 16:**
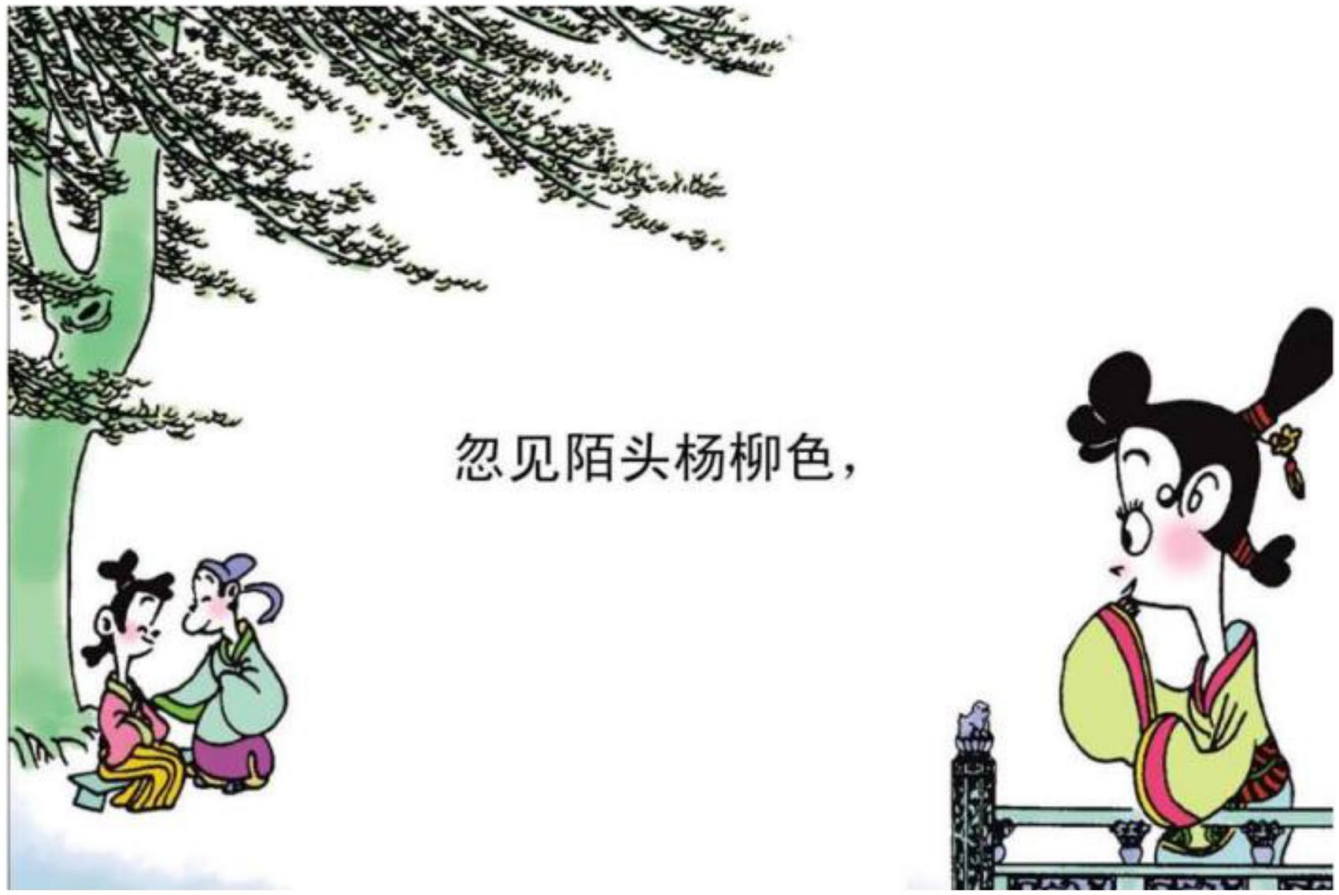
The forth panel on Cai (2014, p. 33). Images reproduced with permission from Shang Dong People Press.

Example 13 shows that the mental process of 见 “see” remains the same in image, representing a lady observing the willow. However, some actions are added in image because a couple are depicted under the willow (see Figure 16). Actually, the lovers are intended to visualize the hidden message in the poem: the vigor of the willow symbolizes the unity of lovers. When the lady sees the willow, the vigor reminds her of her husband who leaves for the battlefield. Hence, the actions here are added to materialize the metaphor of 杨柳色 “willow color”.

The above analysis reveals that actions are used to construct meanings beyond the material processes in poems. They could be deployed to represent attitudes, emotional changes, and metaphors. Through various actions, the comic artist not only makes the poem more dynamic but also uncovers deep messages underlying the poem.

### Functions of verbal processes

The visual process of talking, which is typically realized as speech bubbles, should be considered as a distinct feature of comics. The semiotic translation of various processes toward talking are highly motivated. Hence, these cases, though small in number, are worth a few comments.

The first motivation for using verbal processes in images is to provide verbal explanation of the corresponding line in poems. As speech bubbles are semiotic resources to project what the protagonist says, the comic artist may utilize this resource to interpret or explain the poem. This motivation could be clearly observed in the case below.

14.一片 冰心 在 玉壶。 A patch ice-heart at jade vase.

“A patch of ice-heart is at the jade vase. (A metaphor for being honest and upright).”

15.赢得 青楼 薄幸名 earn brothel name of a fickle.

“Known as fickle， even in the Street of Blue Houses.”

**Figure 17:**
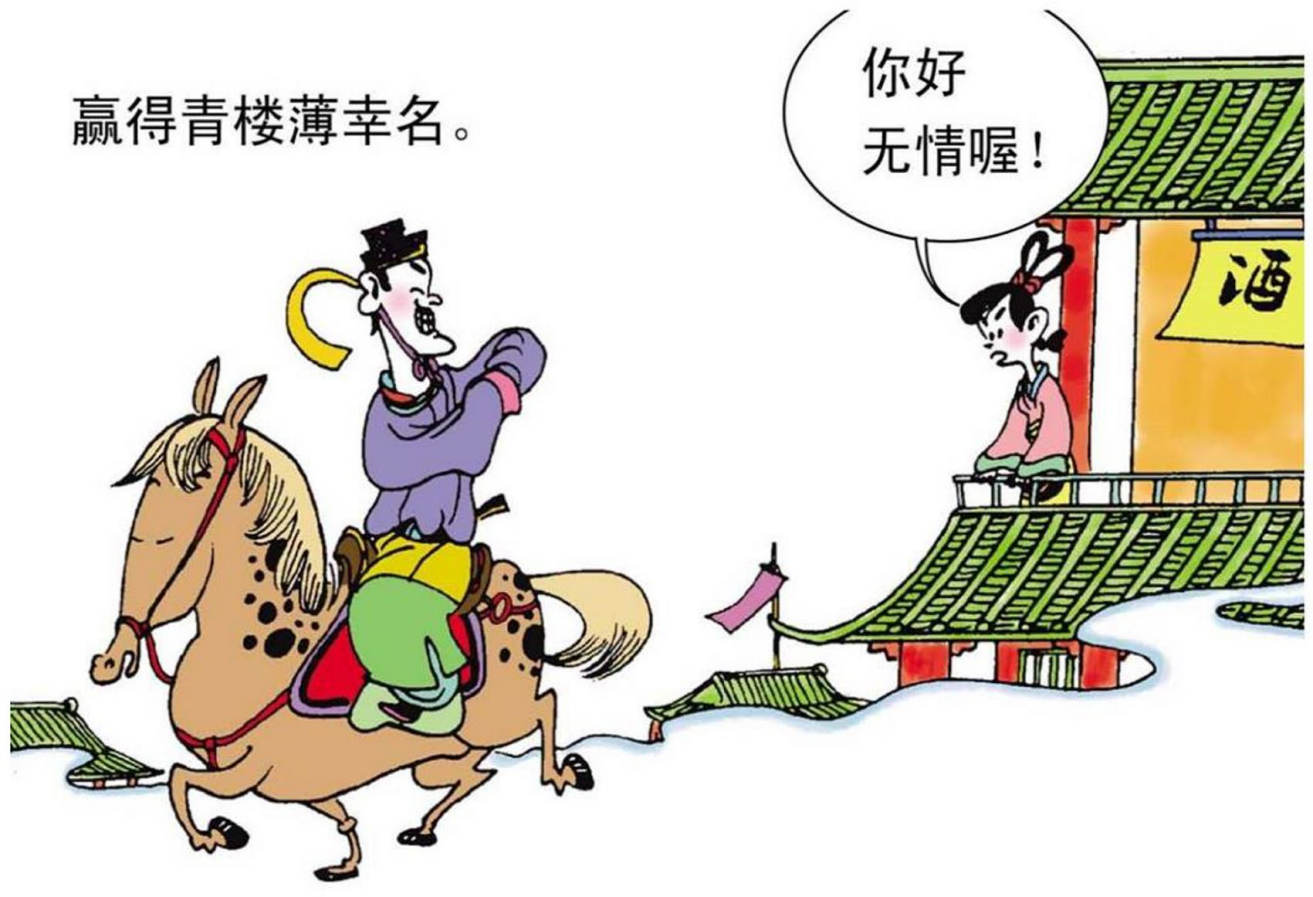
The fifth panel on Cai (2014, p. 132). Images reproduced with permission from Shang Dong People Press.

In example 14, the relational process 在 “at” is intended to construe a metaphor for one’s integrity. This hidden message is disclosed through the verbal processes of the protagonist in the image. The verbiage in the speech bubble can be generally interpreted as “Tell them I am fair and free from corruption.” In Figure 17, the lady says “你好无情哦!(You are such a fickle)”. This is clearly intended to explain the line of poem in example 15. Thus, speech bubbles are often found in some highly sophisticated poems that deserve interpretation.

Another motivation is that the comic artist uses verbal processes to dramatize the poem. Here, dialogs are more imaginative than interpretive. For instance:

16.黄金 然 桂尽 gold already spent.

“Money has all been spent.”

17.人间 能得 几回 闻? Earth can several times hear.

“It is hardly heard on the earth.”

**Figure 18:**
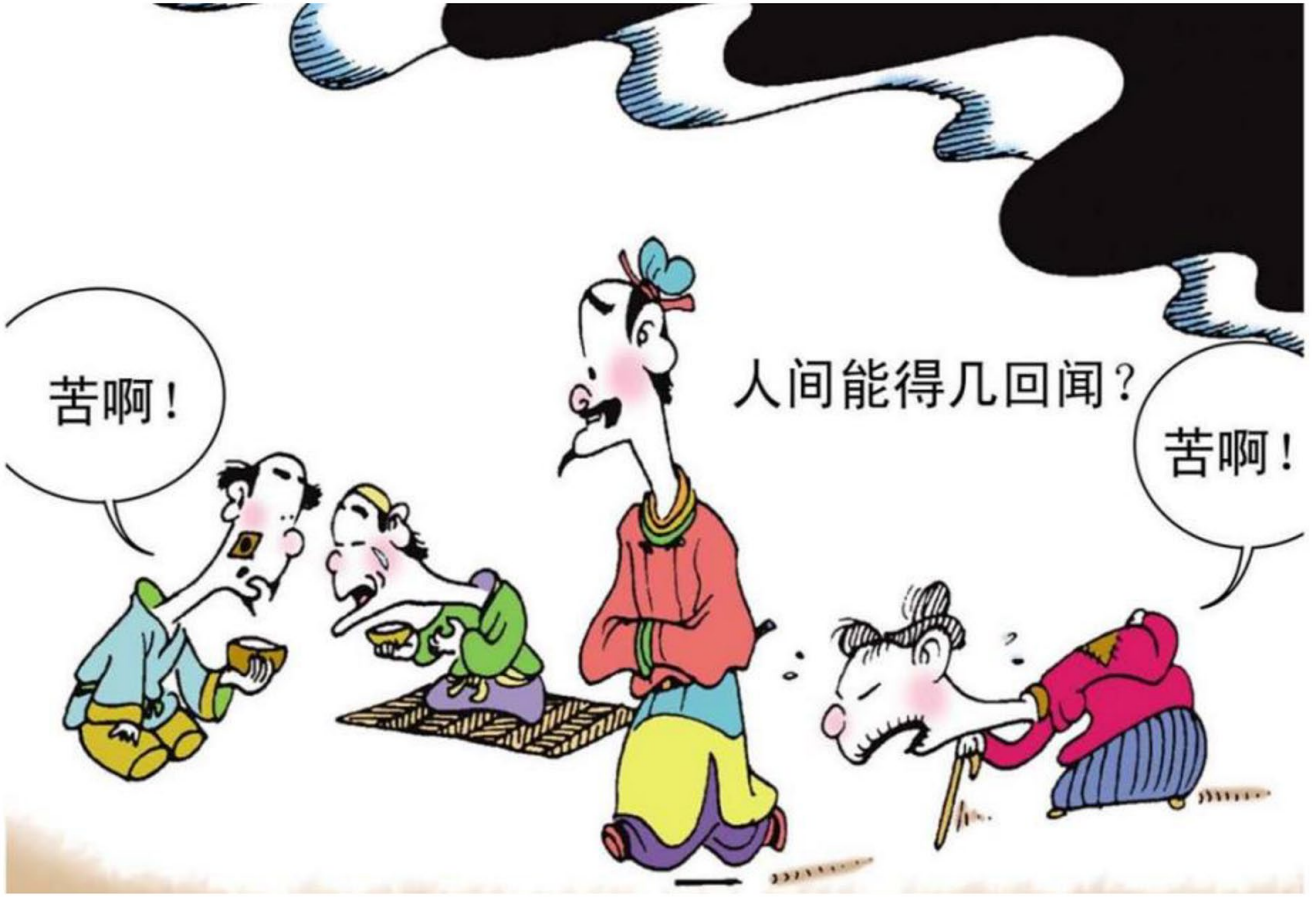
The fifth panel on Cai (2014, p. 66). Images reproduced with permission from Shang Dong People Press.

In example 16, the material process of 尽 “spent” implies that the poet was in financial difficulties. In the image, the layer of meaning is transformed into an imaginative event, in which the protagonist is portrayed as shocked while the vendor says “30 cents each.” This could be considered as dramatization of the financial constraint indicated in the poem. The verbal process of the vendor, together with the mental process of the protagonist, reconstructs the material process of the poem. In example 17, the poem describes the difficult life of the common people in that particular historical period. The verbal process 苦啊 (literally translated as “How bitter!”) is used to dramatize this hidden meaning (see Figure 18).

To conclude, speech bubbles play a distinctly different role in poem comics. They might either help the audience interpret a poem or turn it into a drama. The comic artist of the case under examination is especially adept in managing speech bubbles and sound curves in the context of poems.

## Discussion

Cai’s classic comics has achieved enormous success. His works have not only been the best-sellers of the kind, but also been included in school textbooks in more than 50 institutions as a pilot project in Beijing (Zhang, 2014). Cai has also received keen invitations from many renowned publishing houses to adapt more Chinese traditional classics (see, e.g., Chen, 2017). The analyzes above provide detailed semiotic evidence of how classic poetry has been resemioticized into comic images.

First of all, poetry has been transformed into entertaining stories. Semiotically, various processes in language have been reconstructed as actions in comics. Actions have been portrayed in a sequence that by and large conforms to the “comic grammar” as outlined in Cohn (2013), also see Cohn et al. (2017). Cohn (2013) suggests that a comic strip is usually composed of several common steps such as Establisher, Initial, Peak and Release. These steps, from the semiotic perspective adopted in this study, are mainly realized by various actions. The comic strips in our data also manifest similar patterns. Take Figure 1 as an example. The mental processes of perception in the first panel represent the Establisher of the sequence. Then the actions of bowing in the second panel could be considered as initial. The following two panels show more vibrant actions, formulating the Peak, of the strip. The Release of it is realized by the last panel in which explanation is offered of the poem. The poetry comics would therefore draw on the reader’s understanding of the narrative structures to transform poetry appreciation into dramatization (realized through semiotic shifts toward actions and verbal processes as mentioned in Sections 5.1 and 5.4).

Moreover, we have also noted that many of the behavioral actions on face, such as smiling, laughing, panicking, and crying, are added into the comics while there are no such layers of meanings expressed by the corresponding poetry lines. They can be interpreted using the theoretical framework of visual attitude (Economou, 2009, chapter 4). Some of these actions can be understood as an inscribed positive affect, through which the author has managed to outline a more socially and ecologically harmonious world by depicting the relationship between humans and that between human and nature. Different social relationships such as lovers, couples, king-subordinates, colleagues, friends, etc. could be found across the book. The relationship between human and nature, on the other hand, is constructed through how the protagonist observes the world. In this way, the comic adaptation has served the pedagogical function of poems to arouse the pure emotions toward humans and nature (Yi, 2010, p. 106).

The comic adaptation has also made the traditional poetry more accessible by turning it into images. But images are not necessarily more accessible than language (c.f. Newell, 2017, p. 63–98) so in comics, the images have to be accompanied by poems and explanations in order to be fully understood. Here, we posit that comics increase accessibility of poetry with its particular semiotic resources. Comics, as McCloud (1993, p. 31) argues, could focus our attention on the essential meaning of an idea by eliminating details. On the other hand, the simplified icons in comics call for the reader’s participation and imagination to fill the gutter between the panels (McCloud, 1993, p. 59–61). These characteristics have also been found in our data. First, the part of poem that is more difficult to be understood tends not to be constructed in comics. However, the essential meaning has been preserved by symbolic objects. For instance, metaphors in the poem have been visualized as actions. At the same time, readers have been invited to take the place of the protagonist portrayed in comics to perceive the simplistic objects and circumstances and to imagine the poetic world in the gutter between panels. Comics, in this sense, helps the reader to temporarily get around the incomprehensible part of the poem and forget about the meaning between the lines of poem. Instead, their attention has been shifted, through the semiotic transformation of comics, to the essential meaning and imagery of the poem. When the participatory reading experience is done, the last panel of the comic strip would offer explanation in plain modern Chinese to bring the reader back to the specific meanings of the poem. It is in this sense we argue that comics would alleviate the pressure which the ancient language of poems imposes on the reader. Thus, comics have their unique roles to play in making the Chinese classics more accessible.

Through the intersemiotic translation, the comic artist has also added various elements of traditional culture displaying social etiquettes, conventional clothing, architecture styles, etc. in Tang dynasty. These elements cannot be showcased by a written poem, given its non-visual nature. The social significance of showing these elements should not be under-estimated. Take social etiquettes as an example. Actions such as bowing, toasting, kneeling, etc. are important semiotic resources that the comic artist creates to signal the social order at that time. This is actually a vivid representation of the Confucian thought that focuses on the cultivation of virtue in a morally organized world. Hence these elements, through semiotic processes of action and conceptualization, would infuse the traditional values into the reader.

Despite the additional effects added to the poems, comic images might have translated the poems at the cost of poetry. As American poet Robert Frost put it, “poetry is what gets lost in translation.” Here we argue that poetry is what is lost in intersemiotic translation. If attention were drawn to the visual elements and comic narratives alone, the beauty of poetic meanings and linguistic forms and the imagery therein would be deprived. In this sense, it would be better to treat poetry comics as a gateway to poetic appreciation at the entry level or for the purpose of dissemination.

To sum up, poetry comics have resemioticized the poetic world into an interesting and readable story, which demands greater participation of the reader. In the meanwhile, the visualization of cultural elements helps infiltrate traditional values. Hence, this type of intersemiotic translation has been embraced by the general public and officials.

## Conclusion

Comic adaptation of classics has become one of the most important ways to “inherit and develop the traditional culture” in China. However, there have been relatively few studies which offer any insight into this peculiar type of intersemiotic translation. This paper set out by asking how poetry has been semiotically transformed into comics. Drawing on the transitivity system offered in systemic functional semiotics, we examined the distribution of various types of processes in poems and comics in one of Cai’s books, using UAM image as the annotating tool. Quantitatively speaking, some distinct patterns have been recognized. Mental process is the dominant process in images, thus taking readers to perceive the poetic world. In addition to this, relational and existential processes in the poems were transformed into perceptions, actions, and verbal processes in images. Qualitatively speaking, various processes in comics have not only come to construct the poet’s gaze, build relations, dramatize the poem and visualize the metaphor, but also increase the accessibility of it by inviting the reader to grasp the essential meaning and to fill the gutter between the panels with imagination. More specifically, these processes have woven together to develop a storyline, visualize the metaphor, foreground the protagonists and display various significant cultural elements. Our analyzes provide semiotic account for why the adaptation in question has been accepted so widely in China. And it should be noticed that the dissemination effect might have been achieved at the expense of poetry lost in the entertaining images.

Finally, some limitations of the paper should be noticed. First, only one book has been investigated although the size of data is large enough to reveal some patterns. Future research may compare different versions of poetry comics or poetry paintings such as those drawn by Zikai Feng (丰子恺). Second, we only took process type as the mere indicator for the intersemiotic translation. Other systems such as appraisal and logic-semantic relationships could also be explored for future studies. The poetry comic adaptation could also be studied from a cross-cultural perspective, to see, for example, how the comics are interpreted differently by individuals from western cultures.

## Data availability statement

The original contributions presented in the study are included in the article/supplementary material, further inquiries can be directed to the corresponding author.

## Author contributions

ZZ complied with the multimodal corpus and did the statistics and devoted it to Chapters 1 and 2 and Conclusion. SC was devoted to Chapters 3, 4, and 5. All the authors annotated the data, contributed to the article, and approved the submitted version.

## Funding

This research was supported by the Project “On the Translation and Dissemination of Chinese Classics in Modern English Periodicals (1800–1949)” (Project No.: 17BYY 053) sponsored by the National Social Science Fund of China.

## Conflict of interest

The authors declare that the research was conducted in the absence of any commercial or financial relationships that could be construed as a potential conflict of interest.

## Publisher’s note

All claims expressed in this article are solely those of the authors and do not necessarily represent those of their affiliated organizations, or those of the publisher, the editors and the reviewers. Any product that may be evaluated in this article, or claim that may be made by its manufacturer, is not guaranteed or endorsed by the publisher.
